# Evolution of *Bacillus thuringiensis* Cry toxins insecticidal activity

**DOI:** 10.1111/j.1751-7915.2012.00342.x

**Published:** 2013-01

**Authors:** Alejandra Bravo, Isabel Gómez, Helena Porta, Blanca Ines García-Gómez, Claudia Rodriguez-Almazan, Liliana Pardo, Mario Soberón

**Affiliations:** Instituto de Biotecnología, Universidad Nacional Autónoma de México. Apdo. postal 510-3Cuernavaca 62250, Morelos, Mexico

## Abstract

Insecticidal Cry proteins produced by *Bacillus thuringiensis* are use worldwide in transgenic crops for efficient pest control. Among the family of Cry toxins, the three domain Cry family is the better characterized regarding their natural evolution leading to a large number of Cry proteins with similar structure, mode of action but different insect specificity. Also, this group is the better characterized regarding the study of their mode of action and the molecular basis of insect specificity. In this review we discuss how Cry toxins have evolved insect specificity in nature and analyse several cases of improvement of Cry toxin action by genetic engineering, some of these examples are currently used in transgenic crops. We believe that the success in the improvement of insecticidal activity by genetic evolution of Cry toxins will depend on the knowledge of the rate-limiting steps of Cry toxicity in different insect pests, the mapping of the specificity binding regions in the Cry toxins, as well as the improvement of mutagenesis strategies and selection procedures.

## Introduction

*Bacillus thuringiensis* (Bt) is a Gram-positive bacterium that produces insecticidal proteins as crystal inclusions during its sporulation phase of growth, known as Cry or Cyt toxins, which have been proven to be effective against important crop pests and also against mosquitoes that are vectors of human diseases such as dengue and malaria (Bravo *et al*., 2011). Bt was first discovered in 1901 in Japan by Shigetane Ishiwatari when the causal agent of wilt disease in silk worm (*Bombyx mori*) was isolated. Few years later Bt was rediscovered in Germany by Ernst Berliner from a Mediterranean fluor moth (*Ephestia kuehniella*) (reviewed by Sanahuja *et al*., 2011). The first Bt formulation was developed using the Bt strain isolated by Berliner in 1938. However, the success of Bt as bioinsecticide came with the development of Bt-crops that express the *cry* gene resulting in crops that resist insect attack including borers that were difficult to control with topical Bt-formulations leading to the commercial release of Bt-crops in 1995 (Sanahuja *et al*., 2011). Bt toxins are specific to a limited number of insect species with no toxicity against humans or other organisms (Bravo *et al*., 2011). In 2010, more than 58 million hectares were grown worldwide with Bt-Maize or Bt-cotton (James, 2010).

Many Bt strains that show activity towards Lepidoptera, Diptera, Coleoptera, Hymenoptera, Homoptera, Orthoptera and Mallophaga insect orders have been reported (Schnepf *et al*. 1998). In addition, Bt strains active against nematodes, mites and protozoa have also been isolated (Crickmore *et al*., 1998; Schnepf *et al*., 1998; de Maagd *et al*., 2001; Wei *et al*., 2003). However, still there are many insect pests that show no susceptibility to Cry toxins or that are poorly controlled by the Cry proteins identified so far. On the other hand, a major threat for the use of Cry toxins in transgenic plants is the appearance of insect resistance. Evolution of resistance to Bt-crops in the field has been documented for at least five different insect species (van Rensburg, 2007; Tabashnik *et al*., 2008; Bagla, 2010; Storer *et al*., 2010; Gassmann *et al*., 2011). Therefore, an alternative for the screening and isolation of novel Cry toxin protein in nature, is the *in vitro* genetic evolution of Cry toxins with the aim of enhancing toxicity against specific pests, to kill novel targets or to recover toxicity in the case of the appearance of resistance in the field (Pardo-López *et al*., 2009).

## Natural evolution of Cry toxins

Extensive screening of Bt strains and *cry* gene sequencing has led to the identification of more than 700 *cry* gene sequences (Crickmore *et al*., 2011). These sequences have been classified according to their amino acid sequence identity in at least 70 different *cry* gene groups (Cry1, Cry2… Cry70) where toxins belonging to each Cry group share less than 40% amino acid identity with proteins from other groups (Crickmore *et al*., 1998). Within each group, a capital letter (Cry1A, Cry1B etc) is given when they share less that 70% identity. A small letter (Cry1Aa, Cry1Ab etc) is given when toxins share more than 70% but less than 95% identity. Phylogenetic analysis of Cry protein sequences showed that the whole family of Cry proteins belong to four non-phylogenetically related protein families, the family of three domain Cry toxins (3D), the family of mosquitocidal Cry toxins (Mtx), the family of the binary-like (Bin) and the Cyt family of toxins (reviewed in Bravo *et al*., 2005). Some Bt strains produce additional insecticidal toxins named VIP during vegetative growth, these proteins do not form parasporal crystals, thus were not named Cry toxins. Three VIP toxins have been characterized as VIP1/VIP2, which together compose a binary toxin, and VIP3 (Estruch *et al*., 1996; Warren, 1997).

**Fig. 1:**
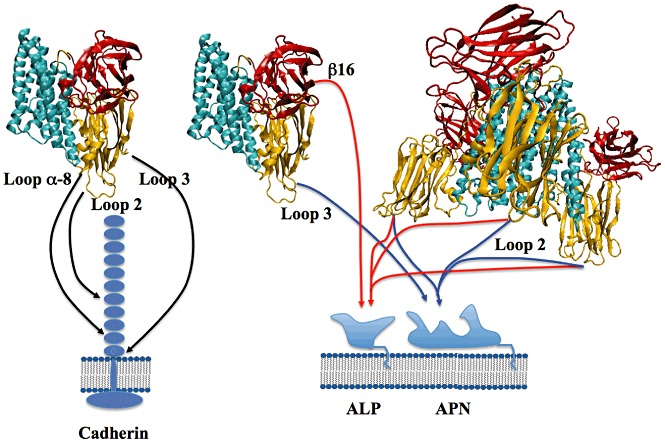
Binding regions of monomeric and oligomeric forms mapped in Cry1Ab toxin to *Manduca sexta* receptors, cadherin, alkaline phosphatase (ALP), and aminopeptidase-N (APN). The monomeric form depicted corresponds to the three-dimensional structure of Cry1Aa (pdb 1CIY) and the oligomeric structure corresponds to Cry4Ba trimeric structure (pdb 1W99).

The 3D family group is the largest group of Cry toxins with over 50 different Cry groups. At least seven 3D-Cry proteins have been crystallized and their three-dimensional structures were solved, Cry1Aa, Cry2Aa, Cry3Aa, Cry3Ba, Cry4Aa, Cry4Ba and Cry8Ea (reviewed in Bravo *et al*., 2011). All 3D-Cry proteins resolved structures show a similar fold, composed of three domains despite the fact that some of these proteins share very little amino acid sequence identity (less than 20%). Domain I is a seven α-helix bundle comprised by six amphipatic helices surrounding the hydrophobic helix α-5. This domain has been shown to be involved in toxin oligomerization, membrane insertion and pore formation. Domain II is composed of eleven beta sheets with exposed loop regions involved in binding to specific larval midgut proteins while domain III is a beta sandwich that is also involved in receptor recognition. Thus domains II and III are the specificity determinant domains of Cry toxins (Bravo *et al*., 2011) (Fig. 1). This group of 3D-Cry proteins is characterized by their production during sporulation of the bacteria as protoxins, with some members producing large protoxins of 130 kDa, such as the Cry1Aa protoxin, while other members are synthesized as short protoxins of 65–70 kDa, such as the Cry11Aa protoxin. In the case of large protoxins, they are processed by insect midgut proteases loosing half of the protein at the C-terminal end, approximately 600 amino acids. The large protoxins are also processed at the N-terminal end, where 20–50 amino acid residues were cleaved out, depending on the toxin. The short protoxins are only processed at the N-terminal end (de Maagd *et al*., 2001). The proteolytical activation of both, large or short Cry protoxins resulted in a protease resistant core of approximately 60 kDa that is biological active and is comprised by the three-dimensional structure (de Maagd *et al*., 2001).

The phylogenetic analysis of the 3D-Cry protein family revealed a different topology when the protoxin or the mature toxin protein fragments were analysed (Bravo, 1997; Crickmore, 2000; de Maagd *et al*., 2001). Cry toxins are classified by the amino acid sequence similarity of protoxin sequences. Phylogenetic analysis of toxin fragments revealed different evolutionary relationships of certain Cry toxins than the analysis of protoxin sequences (Bravo, 1997; Crickmore, 2000). For instance, Cry9Aa toxin fragment shows no evolutionary relationship with Cry9Ba or Cry9Ca toxin fragments indicating that the high sequence identity at the C-terminal protoxin fragment was responsible for clustering these toxins together (Bravo, 1997; Crickmore, 2000). The phylogenetic analysis of toxin fragments revealed interesting clustering of Cry proteins accordingly with their different insect specificity. However, some exceptions were found as exemplified by Cry1B, Cry1I that show toxicity against lepidopteran insects that clustered together with Cry3, Cry7 and Cry8 that are toxic to coleopteran insects (Bravo, 1997; Crickmore, 2000). This observation suggests that Cry1B and Cry1I proteins may have toxicity against some coleopteran as was latter shown for Cry1B toxin (López-Pazos *et al*., 2009). Thus, the phylogenetic relationships of whole protoxin did not revealed how Cry toxins evolved insect specificity. Interestingly, the analysis of the evolutionary relationships of single domains revealed that domains I and II co-evolved as the different toxins showed similar clustering when domain I or domain II sequences were analysed independently (Bravo, 1997; Crickmore, 2000). Interestingly, phylogenetic analysis of domain III sequences revealed several examples of domain III swapping among different toxins. For example, Cry1Ac and Cry1Bd share a similar domain III while Cry1Be, Cry1Cb and Cry1Eb share a related domain III amino acid sequence. Thus domain III swapping among different Cry toxins is likely to be an active evolutionary process for determining insect specificity (Bravo, 1997; de Maagd *et al*., 2001).

The analysis of adaptive evolution revealed several residues of 3D-Cry toxins that are under positive selection (Wu *et al*., 2007). Positive selection favours the retention of mutations that are beneficial to an individual or a population. Twenty-four residues were identified to be under positive selection and most of them located either in domain II loop regions or domain III, suggesting that these amino acid regions are likely to be involved in receptor recognition. Based on this result, it was proposed that the high divergence found in these regions could promote rapid evolution to their targets insect receptors (Wu *et al*., 2007). It was also proposed that the diversity of Cry toxins found in nature is the result of two fundamental evolutionary process, the independent evolution of the three structural domains with domain II and domain III regions under positive selection for insect receptor recognition and domain III swapping. These evolutionary processes had led to the selection of proteins with similar mode of action but with different insect specificity.

## Mode of action of 3D-Cry toxins

As mention previously, different members of the 3D-Cry toxins share a similar three-dimensional fold suggesting that they share a similar mode of action. 3D-Cry toxins are recognized as pore forming toxins that kill larval epithelium midgut cells by causing an osmotic shock leading to cell lysis. To induce the pore formation of 3D-Cry toxins, the parasporal crystals have to be ingested by susceptible larvae, solubilized by the pH conditions of the insect gut, alkaline in the case of lepidopteran and dipteran insects and acidic in the case of coleopteran, and activated by midgut proteases to yield the three-dimensional resistant core of the activated toxin. In the case of Cry1A toxins that are active against lepidopteran insects, it has been shown that Cry1A toxins undergo a sequential binding mechanism with glycosyl-phosphatidyl-inositol anchored proteins such as alkaline phosphatase (ALP) or aminopeptidase-N (APN) and cadherin-like protein resulting in the formation of a pre-pore oligomeric structure that is proficient in membrane insertion and pore formation (Bravo *et al*., 2011). Receptor recognition by Cry toxins has been recognized as a key step of Cry toxicity that is fundamental for insect specificity (Schnepf *et al*., 1998; Bravo *et al*., 2011). However, protoxin activation by insect proteases has been shown to be also a limiting step either due to un-efficient toxin activation or rapid proteolytic degradation that in some cases has been shown to determine insect specificity (de Maagd *et al*., 2001). For instance, Cry7Aa is active against the coleopteran Colorado potato beetle only after *in vitro* solubilization and trypsin activation of the protoxin, suggesting that solubilization and activation of Cry7Aa is a limiting step of the insecticidal activity of this Cry protein (Lambert *et al*., 1992).

The mode of action of Cry1Ab toxin has been described to some detail in the tobacco hornworm (*Manduca sexta*) larvae. It is proposed that Cry1Ab binds with low affinity (*Kd* 100–200 nM) to glycosyl-phosphatidyl-inositol anchored ALP or APN receptors. This binding step is believed to concentrate the monomeric toxin in the surface of the brush border membrane. Following this binding step Cry1Ab binds with high affinity (*Kd* 1 nM) to cadherin; this binding step facilitates the proteolytic removal of helix α1 of domain I, inducing toxin oligomerization. Cry1Ab toxin oligomers gain binding affinity to both ALP and APN (*Kd* 0.6 nM) and this final binding step facilitates oligomer membrane insertion and pore formation (reviewed in Bravo *et al*., 2011). However, a different model of the mode of action of Cry toxins proposed that binding to cadherin is sufficient to trigger an intracellular signal transduction pathway that leads to cell death without the involvement of oligomer formation nor pore formation (Zhang *et al*., 2006). Nevertheless, as discussed below, the construction of modified Cry toxins that skip cadherin interaction has shown that binding to cadherin is not sufficient for toxicity (Soberón *et al*., 2007). The regions involved in the binding of Cry1Ab monomeric and oligomeric forms to these receptor molecules were mapped and shown to include domain II loop regions and exposed surface of domain III (Gómez *et al*., 2006; Pacheco *et al*., 2009; Arenas *et al*., 2010). Cry1Ab monomeric toxin binds ALP or APN through domain II loop 3 and domain III β16. In contrast, the oligomeric form of the Cry1Ab toxin binds ALP or APN through domain II loop 2 (Pacheco *et al*., 2009; Arenas *et al*., 2010). Binding to cadherin is mediated by three binding sites that include domain II loop α8, loop 2 and loop 3 (Gómez *et al*., 2003; 2006). Figure 1 shows the binding epitopes of Cry1Ab toxin that are involved in receptor recognition.

## *In vitro* evolution of Cry toxin insecticidal activity

### Proteolytic activation of Cry toxins

As mentioned previously activation by insect midgut proteases could be a limiting step of Cry toxicity in different insect species. In the case of Cry3Aa that has insecticidal activity against coleopteran larvae like Colorado potato beetle (*Leptinotarsa decemlineata*) it shows very low toxicity against Western corn rootworm (*Diabrotica virgifera virgifera*). The low toxicity of Cry3Aa against Western corn rootworm was proposed to be due to the low solubility of the protease activated Cry3Aa that yield a 67 kDa fragment. However, activation with chimiotrypsin was shown to increase the yield of a fully processed 55 kDa Cry3A fragment that showed increased solubility and toxicity (Carrrol *et al*., 1997). The 55 kDa form was shown to be nicked at the α3-α4 domain I loop region (Carrrol *et al*., 1997). The introduction of a chymiotrypsin/cathepsin G proteolytic site in the Cry3Aa α3-α4 loop (named mCry3Aa) resulted in increased yields of the 55 kDa form and increased toxicity towards Western corn rootworm (Walters *et al*., 2008). The increased toxicity of mCry3Aa also correlated with increased solubility of the 55 kDa processed form and also with the specific binding of the processed 55 kDa to Western corn root worm brush border membrane vesicles (BBMV) (Walters *et al*., 2008). Interestingly, mCry3Aa showed a similar insecticidal activity towards Colorado potato beetle larvae as Cry3Aa indicating the engineered protease site in mCry3Aa broadened the insecticidal activity rather changing its insect specificity. mCry3Aa has been expressed in transgenic maize and shown to be effective in controlling Western corn rootworm (Hibbard *et al*., 2011).

Appearance of insect resistance threatens the use of Cry toxins in transgenic plants (Tabashnik *et al*., 2008). In different lepidopteran insect colonies it has been shown that resistance to Cry1Ab or Cry1Ac toxins is linked to mutations in the cadherin gene (reviewed in Bravo and Soberón, 2008). Cadherin binding is a limiting step of Cry1A toxins action as it facilitates further proteolytic processing of the toxin removing helix α1 necessary for toxin oligomerization. Cry1Ab and Cry1Ac toxins that were genetically modified to delete helix α1 (Cry1AbMod or Cry1AcMod) were shown to form oligomers *in vitro* when activated with proteases in the absence of cadherin protein in contrast to native toxins that only formed the oligomeric structure when activated in the presence of a cadherin binding site (Soberón *et al*., 2007). Cry1AbMod and Cry1AcMod were shown to counter resistance of the Pink bollworm (*Pectinophora gossypiella*) Cry1Ac resistant colony linked to mutations in the cadherin gene (Soberón *et al*., 2007). This result suggested that Cry1AMod toxins have the potential to counter insect resistance when resistance is linked to mutations affecting cadherin expression. Interestingly, a follow-up study analysed the insecticidal activity of Cry1AbMod and Cry1AcMod against seven different lepidopteran insect resistant colonies that in some cases were not linked to mutations affecting cadherin expression (Tabashnik *et al*., 2011). These insect colonies included a laboratory selected tobacco budworm (*Heliothis virescens*) Cry1Ac resistant colony whose resistance was recently shown to be genetically linked to a mutant allele of an ABC transporter (ABCC2) (Gahan *et al*., 2010). The ABCC2 mutation affected the binding of Cry1Ab and Cry1Ac to BBMV. It was proposed that the ABCC2 protein could facilitate oligomer membrane insertion (Gahan *et al*., 2010). Also, the toxicity analysis of Cry1AMod toxins included two field evolved Cry1Ac resistant colonies of Diamondback moth (*Plutella xylostella*) and the cabbage looper (*Trichoplusia ni*) that were recently shown to be also linked to mutations in the ABCC2 transporter (Baxter *et al*., 2011). The data revealed that Cry1AMod also counters resistance in these two insect resistant lines and also in tobacco budworm resistant colony that contains both the ABCC2 and cadherin mutant alleles. Interestingly, Cry1AbMod and Cry1AcMod were not very effective against the tobacco budworm single mutant cadherin or ABCC2 insect colonies (Tabashnik *et al*., 2011). It is important to mention that Cry1AbMod and Cry1Ac toxins were not very effective against the susceptible line of tobacco budworm, showing an important reduction in toxicity, suggesting that the low efficacy of Cry1AMod toxins against insect lines with low resistance ratios, such as the tobacco budworm colonies affected only in cadherin gene or in ABCC2 gene, could be due to the reduce activity of Cry1AMod toxins when compared with the native Cry1A toxin. These results suggest that the mode of action of Cry1A toxins is more complex than explained above and will include additional proteins such as ABCC2 transporter and that although that Cry1AMod toxins may have lower efficacy against some insect pests, they have the potential to counter resistance based on different mechanisms (Tabashnik *et al*., 2011). The basis of the low efficacy of CryMod toxins against particular insect pests remains to be analysed. This could lead to the improvement of Cry1AMod in the future.

### Domain III swapping

As mentioned above domain III swapping has been recognized as a natural mechanism involved in the evolution of Cry toxins. *In vitro* domain III swapping among different Cry toxins have resulted in some cases in hybrid toxins with improved toxicities against certain insect species (Bosch *et al*., 1994). One of the first examples was the construction of a hybrid toxin containing domains I and II from Cry1Ab toxin and domain III of Cry1C that showed more than sixfold higher toxicity against beet armyworm (*Spodoptera exigua*) compared with Cry1C (de Maagd *et al*., 2000). Improvement of toxin activity by domain III swapping was also shown for the coleopteran Colorado potato beetle. In this case both Cry1Ba and Cry1Ia showed low toxicity against Colorado potato beetle. Construction of a hybrid toxin consisting of domains I and II of Cry1Ia and domain III of Cry1Ba showed three- and sevenfold higher insecticidal activity against Colorado potato beetle than the parental toxins Cy1Ia and Cry1Ba respectively (Naimov *et al*., 2001). An interesting example of toxin improvement by domain III swapping was the construction of a hybrid toxin containing domains I and II from Cry3Aa and domain III from Cry1Ab (eCry3.1Ab). eCry3.1Ab was shown to be toxic to Western corn rootworm in contrast to Cry3Aa and Cry1Ab that showed no toxicity to this insect (Walters *et al*., 2010). Interestingly, Cry1Ab is a lepidopteran specific toxin but its domain III could improve the toxicity of a coleopteran specific toxin such as the Cry3Aa. eCry3.1Ab expressed in transgenic maize was shown to be effective in controlling Western corn rootworm (Hibbard *et al*., 2011). Overall, these results show that domain III swapping could be an interesting strategy to improve toxicity of Cry toxins or to create novel hybrid toxins with toxicity against pests that show no susceptibility to the parental Cry toxins. Strategies for shuffling the three different domains among large numbers of *cry* genes (Knight *et al*., 2004) and high through output bioassay screening methods are likely to provide novel Cry toxins with improved or novel toxicities.

### Domain II and Domain III mutations with enhanced insecticidal activity

Exposed loop regions in domain II have been shown to be important determinants of insect specificity (Bravo *et al*., 2005). Cry4Ba shows no toxicity against *Culex sp* while Cry4Aa is active against this mosquito species (Abdullah *et al*., 2003). Introducing domain II loop 3 Cry4Aa amino acid sequence into loop 3 of domain II of Cry4Ba resulted in a mutant toxin (4BL3GAV) with toxicity against *Culex sp*, retaining its insecticidal activity against *Aedes aegypti* (Abdullah *et al*., 2003). In the same line of ideas, the lepidopteran Cry1Aa toxin was engineered in domain II loop regions to mimic the Cry4Ba mutant 4BLGAV showing that the engineered Cry1Aa (1AaMosq) gained activity against *Culex pipiens* larvae, although the activity was three orders of magnitude lower than 4BLGAV (Liu and Dean, 2006). These results suggest that domain II loop swapping could provide a strategy for improving or changing the specificity of Cry toxins.

Site-directed mutagenesis of domain II loop sequences has in some cases resulted in mutant toxins with increased insecticidal activity. The first example of domain II loop mutants with increased insecticidal activity was Cry1Ab toxin where mutations in loop 2 resulted in higher insecticidal activity against Gypsy moth (*Limantria dispar*) (Rajamohan *et al*., 1996). A single Cry1Ab mutation in loop 2, N372A, or a triple loop 2 mutant in residues N372A, A282G and L283S showed 8- and 36-fold higher toxicity to Gypsy moth larvae respectively. Interestingly, the increased insecticidal activity correlated with increased binding affinities to BBMV isolated from Gypsy moth (Rajamohan *et al*., 1996). Similarly, it was shown that mutations of domain II loop regions in the coleopteran Cry3Aa resulted in enhanced toxicity to yellow mealworm (*Tenebrio molitor*) (Wu *et al*., 2000). A triple domain II loop 1 mutant R345, Y350F, Y351F showed tenfold higher toxicity to yellow mealworm than Cry3Aa and twofold higher toxicity against Colorado potato beetle that correlated with twofold higher binding affinity to Colorado potato beetle BBMV (Wu *et al*., 2000). These results show that domain II loop regions are key binding regions of Cry toxins that are suitable targets for mutagenesis and selection of Cry toxins with improved insecticidal properties.

In the case of domain III, there are just few examples of mutations in two different exposed loop regions with some mutants showing a moderate non-significant increase in toxicity against different insect species (Shu *et al*., 2007; Xiang *et al*., 2009; Lv *et al*., 2011; Shan *et al*., 2011). Nevertheless, there are few studies that have mapped the domain III binding epitopes with ALP or APN receptors (Atsumi *et al*., 2005; Gómez *et al*., 2006; Arenas *et al*., 2010). Mutagenesis of these domain III binding regions is likely to provide means for increasing Cry toxins insecticidal activity such as domain III swapping that has been shown to create novel toxins with improved toxicities (de Maagd *et al*., 2000).

### Other mutations in Cry toxins with enhanced insecticidal activity

**Fig. 2:**
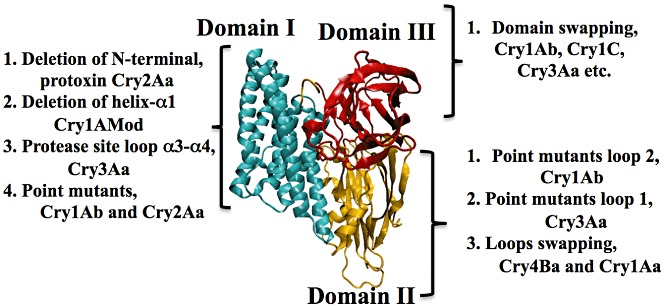
Representation of Cry toxin regions where mutations enhanced insecticidal activity in different Cry toxins. The three-dimensional structure of Cry1Aa toxin (pdb 1CIY) is depicted.

Besides the domain I mutations introducing protease cleavage sites described above, two other modifications in domain I of Cry1Ab or Cry2Aa have shown to increase insecticidal activity. Cry1Ac helix α5 mutant V171C was shown to have 25-fold higher insecticidal activity against Gypsy moth without affecting its toxicity to the tobacco hornworm (Alzate *et al*., 2010). The increased toxicity of Cry1Ac V171C was proposed to be due to a higher unfolding rate allowing the more rapidly partitioning of the toxin into the membrane (Alzate *et al*., 2010). In the case of Cry2Aa, two modifications in domain I resulted in a Cry2Aa mutant with four- to six-fold higher toxicity against cotton leaf worm (*Spodoptera litura*), cotton bollworm (*Helicoverpa armigera*) and black cutworm (*Agrotis ipsilon*) (Mandal *et al*., 2007). The first modification that enhanced threefold the toxicity of Cry2Aa, consisted in the deletion of the first 42 amino acid residues at the N-terminal end. Cry2Ab three-dimensional structure revealed the first 49 amino acids precedes domain I helix α1 and these residues are normally cleaved out during protease activation of Cry2Ab protoxin. This N-terminal fragment was shown to occlude a domain II hydrophobic patch proposed to be involved in receptor interaction (Morse *et al*., 2001). Thus, the proteolytic cleavage of the N-terminal protoxin fragment could be a rate-limiting step that is avoided with the 42 amino acid deletion (Mandal *et al*., 2007). Two additional point mutations in Cry2Aa helix α1 residues K63F and K64P were introduced based on enhancing the hydrophobic nature of a putative transmembrane region identified *in silico* (Mandal *et al*., 2007). Finally, a threefold increased in the insecticidal activity of Cry3Aa against Asian longhorn beetle (*Anoplophora glabripennis*) was achieved by fusion of an eight amino acid residue peptide that was shown to specifically bound longhorn beetle midgut Cx-cellulase (Guo *et al*., 2012). It was proposed that the fused peptide increased the toxin retention in the gut by binding to the gut cellulase (Guo *et al*., 2012). Figure 2 shows the three-dimensional structure of Cry1Aa toxin highlighting the regions where modifications had led to increased insecticidal activity in different Cry toxins.

## High through output systems for evolution of Cry toxins

As indicated above, the improvement of the insecticidal activity by site-directed mutagenesis of the binding epitopes found in domains II and III has a lot of potential for selection of Cry toxins with improved activity against different insect pests as shown by the different examples of Cry toxins with modifications in these amino acid regions that have resulted in toxins with improved insecticidal properties. However, those examples have been the result of analysing few mutants in different insect species but not from a high through output system that could detect improved mutants from a large pool of variants. Gene shuffling was reported to be a useful method for the generation of *cry* gene mutant libraries to select improved variants with increased insecticidal activity. The first example was the evolution of Cry1Ca protein with increased insecticidal activity against fall armyworm. Improved Cry1Ca variants were detected by bioassay screening where mutants showing more than sixfold higher toxicity against tobacco budworm than the parental Cry1Ca toxin were selected (Lassner and Bedbrook, 2001). The regions that resulted in the enhanced insecticidal activity of these Cry1Ca proteins were not reported (Lassner and Bedbrook, 2001). This last approach is likely to provide better Cry mutant toxins as they are selected from many variants. Nevertheless, the identification of improved variants by bioassays is a challenging task when many Cry toxin variants are screened. Thus, a desirable method should be a method that screens Cry mutant libraries for binding to BBMV from the selected insect or to receptor molecules purified from the target insect. In the latter case, it will be desirable to restrict the mutagenesis to the Cry toxin binding regions that are important for binding with specific receptor molecules. Below we will discuss recent work showing that gene shuffling combined with a binding screening selection using phage display could lead to selection of improved Cry toxins.

### Phage display

Phage display allows a rapid selection of variants with improved binding characteristics (Azzazy and Highsmith, 2002; Mullen *et al*., 2006). For this purpose, the foreign protein DNA sequence is fused to a coat protein gene enabling the fusion protein to be displayed on the surface of the phage that can be then screened by enabling the phage to interact with ligands, a process known as biopanning. Filamentous M13 phage libraries are constructed by fusing the protein of interest with a capsid protein such as capsid proteins p3 or p8. The fusion protein can be incorporated to the phage genome or into molecular vectors called phagemids. Phagemid vectors contain a coat protein gene usually with a polilinker site for protein fusion, an antibiotic resistance gene as ampicillin and are capable of replicating in *Escherich coli* cells due to a plasmid replication site and be packed into the phage particle due to a M13 DNA sequence that is recognized by the packaging machinery when a helper M13 phage is used to infect *E. coli* cells harbouring the phagemid constructs (Azzazy and Highsmith, 2002; Mullen *et al*., 2006). To display the fusion protein, it has to be translocated to the host periplasm by a leader-peptide sequence while a helper phage provides all the necessary components for phage assembly. This is a powerful technique as the selected phages maintain a physical link between the displayed protein (phenotype) and the encoding gene (genotype) and further mutagenesis and selection by biopanning allows an *in vitro* high through output molecular evolution of proteins (Droge *et al*., 2003).

Cry1A toxins have been displayed in three different phages M13, T7 and λ but, as discussed below, these systems have shown not to be optimal systems for displaying Cry1A toxins (Marzari *et al*., 1997; Kasman *et al*., 1998; Vílchez *et al*., 2004; Pacheco *et al*., 2006. M13 phage-display systems have an intrinsic problem in displaying big proteins as fusion proteins have to be transported into the *E. coli* periplasm where phage assembly occurs. The first display of Cry1Aa toxin in M13 (Marzari *et al*., 1997; Kasman *et al*., 1998), showed important deletions in the displayed fused toxin protein (Marzari *et al*., 1997). Nevertheless, in another report it was shown that Cry1Ac was displayed in M13 showing toxicity to tobacco budworm larvae, but the displayed Cry1Ac protein did not bind to functional APN receptor *in vitro* suggesting structural constraints of the displayed toxin (Kasman *et al*., 1998). Later it was shown that phages λ and T7 could be much better systems to display Cry1A toxins as the assembling of phage particles in both systems occurs in the cytoplasm of bacterial cells, allowing the display of big proteins (Vílchez *et al*., 2004; Pacheco *et al*., 2006). In the case of λ phage, the Cry1Ac protein was fused with the capsid protein D and displayed on the surface of phage particles. The displayed Cry1Ac toxin retained similar toxicity as the wild-type toxin to tobacco hornworm and the capacity to interact with the APN receptor (Vílchez *et al*., 2004). In another report the *cry1Ac* gene was fused to the 3′ end of the T7 10B capsid protein gene and the chimeric protein was displayed on the surface of T7 phage. The T7-Cry1Ac binds Cry1Ac-receptors and BBMV isolated from tobacco hornworm and retained toxicity against tobacco hornworm larvae, suggesting the successful display of Cry1Ac toxin in T7 phages (Pacheco *et al*., 2006). Nevertheless, a problem with both λ and T7 displaying systems is that for displaying the fusion protein both systems rely on *in vitro* packaging systems that under the best scenario allow for the production of up to 10^7^ recombinant phage particles mg^−1^ of DNA, making the construction of libraries with large number of variants inefficient.

Despite the problems mentioned above on the efficiency to display certain Cry toxins in phage particles, at least three successful examples of selection of improved variants of Cry toxins selected by biopanning of phage display libraries have been described (Ishikawa *et al*., 2007; Craveiro *et al*., 2010; Oliveira *et al*., 2011). The first example was the display of Cry1Aa toxin using T7 phage system. A library of mutants in domain II loop 2 region was constructed and used to recover toxin variants with increased binding affinity to a silkworm (*Bombyx mori*) cadherin fragment. After five rounds of selection using cadherin coated magnetic beads, a domain II loop 2 mutant with apparent similar binding affinity to cadherin, and a fourfold higher insecticidal activity against silkworm larvae than Cry1Aa was selected (Ishikawa *et al*., 2007). The two other examples relied on the construction of Cry toxin library variants by gene shuffling, on the display on M13 of these libraries and selection of improved variants by biopanning against BBMV from the selected target insect. The sugarcane giant borer (*Telchin licus licus*) is not susceptible to the known Cry toxins including Cry1Ia that was shown to be toxic to fall armyworm (*Spodoptera frugiperda*) (Craveiro *et al*., 2010). A library of Cry1Ia variants created by gene shuffling was cloned into a phagemid vector and display in the M13 phage. This library was used to select better Cry1Ia binders to sugarcane giant borer BBMV. Four variants with significant insecticidal activity against sugarcane borer were recover and shown to contain single point mutations in domains I and III (Craveiro *et al*., 2010). Finally, a similar strategy was used for selection of Cry8Ka mutants with increased insecticidal activity against cotton boll weevil (*Anthonomus grandis*). Cry8Ka gene was identified as a toxin showing moderate toxicity against cotton boll weevil. Gene shuffling and the selection of improved Cry8Ka binders against cotton boll weevil BBMV resulted in a mutant (Cry8Ka5) with threefold higher toxicity (Oliveira *et al*., 2011). Cry8Ka5 showed a 16 amino acid deletion in the N-terminal end and six additional amino acid changes, one located in what was predicted as domain II loop 3 amino acid region (Oliveira *et al*., 2011).

## A general strategy for evolution of Cry toxin insecticidal activity

**Fig. 3:**
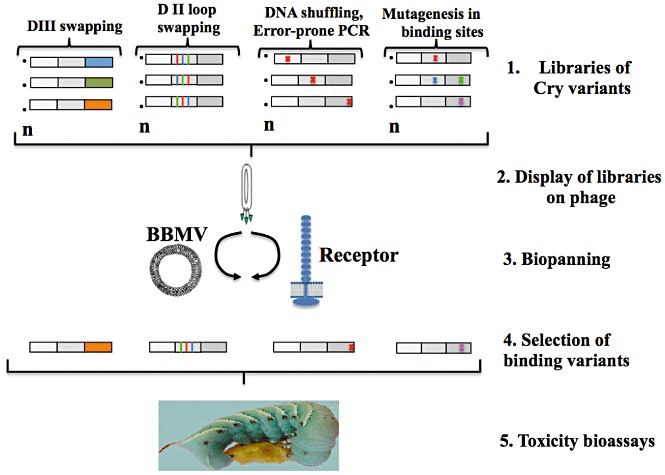
General strategy for *in vitro* evolution of toxicity of Cry toxins. Five steps are proposed for *in vitro* evolution of Cry toxins, 1. Construction of gene libraries with Cry variants obtained by different mutagenesis strategies (prone PCR, gene shuffling, domain III swapping, domain II loop 2 swapping and mutagenesis of receptor binding regions); 2. Display of gene libraries on phage; 3. Biopanning of phage display libraries using brush border membrane vesicles of insect of interest or purified receptors (cadherin is shown as example); 4. Selection of variants with improved binding characteristics; 5. Toxicity assays against the target insect to select Cry toxins with improved insecticidal activity.

We have revised several examples of evolved Cry toxins with improved performance in controlling different insect pests. Some of these Cry mutants show novel insecticidal activity, others improved toxicity against a specific target and others were shown to be active against resistant insects to Cry toxins. Nevertheless, as pointed before, most of these Cry mutants were the result of analysing few mutants in different insect species but not from a high through output system that could detect improved mutants from a large number of variants. We believe that the evolution of Cry toxicity using high through output systems is likely to provide toxins that will perform better in controlling insect pests. In Fig. 3 we propose a general strategy for *in vitro* evolution of toxicity of Cry toxins. The rational behind this strategy is that Cry toxin mutants with improved binding affinities to either BBMV from the target insect or isolated insect Cry-binding proteins, will provide mutants that are likely to show enhanced insecticidal activity. The first step is the construction of *cry* gene library of variants that could then be screened for binding to BBMV or toxin-receptors from the target insect. Several methods for creating variability could be exploited depending on the binding selection procedure. Using general mutagenesis strategies as gene shuffling or prone PCR can explore the whole gene *cry* sequence including domain I. As discussed earlier, there are examples of domain I mutations that enhance Cry toxicity presumably by enhancing membrane partioning into the membrane (Mandal *et al*., 2007; Alzate *et al*., 2010). Gene libraries could also be created by shuffling domain III among different Cry toxins or by shuffling domain II loop regions that are likely to provide Cry toxins with improved toxicity or altered specificity. Finally, gene libraries could be created by mutation of receptor binding epitopes like domain II loop regions or residues of domain III β16. In the second step the *cry* gene libraries are cloned into phagemid vectors for the display of Cry mutants in the phage particles. The third step is the screening of libraries by biopanning against BBMV or pure receptor molecules. The fourth step is the selection of Cry mutants with enhanced binding to BBMV or receptors. Finally, the fifth step is the determination of toxicity of Cry toxins with improved binding against the target insect. Improved variants could be the substrate for additional mutagenesis, binding selection and bioassays.
